# Optofluidic device for the quantification of circulating tumor cells in breast cancer

**DOI:** 10.1038/s41598-017-04033-9

**Published:** 2017-06-16

**Authors:** Eric Pedrol, Manuel Garcia-Algar, Jaume Massons, Moritz Nazarenus, Luca Guerrini, Javier Martínez, Airan Rodenas, Ana Fernandez-Carrascal, Magdalena Aguiló, Laura G. Estevez, Isabel Calvo, Ana Olano-Daza, Eduardo Garcia-Rico, Francesc Díaz, Ramon A. Alvarez-Puebla

**Affiliations:** 1 Física i Cristal•lografia de Materials i Nanomaterials and EmaS. Universitat Rovira i Virgili, Carrer Marcel•lí Domingo 1, 43007 Tarragona, Spain; 2 Departamento de Química Física e Inorgánica and EmaS, Universitat Rovira i Virgili, Carrer Marcel•lí Domingo 1, 43007 Tarragona, Spain; 3 Fundacion de Investigacion HM Hospitales, San Bernardo 101, 28015 Madrid, Spain; 4 Centro Integral Oncologico Clara Campal (CIOCC), Oña 10, 28050 Madrid, Spain; 5 Department of Medical Oncology, Hospital Universitario HM Torrelodones, Castillo de Olivares s/n, 28250 Torrelodones, Spain; 6 School of Medicine, San Pablo CEU, Calle Julián Romea, 18, 28003 Madrid, Spain; 7 ICREA, Passeig Lluís Companys 23, 08010 Barcelona, Spain

## Abstract

Metastatic cancer patients require a continuous monitoring during the sequential treatment cycles to carefully evaluate their disease evolution. Repetition of biopsies is very invasive and not always feasible. Herein, we design and demonstrate a 3D-flow focusing microfluidic device, where all optics are integrated into the chip, for the fluorescence quantification of CTCs in real samples. To test the chip performance, two cell membrane targets, the epithelial cell adhesion molecule, EpCAM, and the receptor tyrosine-protein kinase, HER2, are selected. The efficiency of the platform is demonstrated on cell lines and in a variety of healthy donors and metastatic-breast cancer patients.

## Introduction

Prognosis and assessment of breast cancer treatment are established at the time of diagnosis by pathologic examination, immunohistochemistry, and molecular biology after core needle biopsy or surgical specimen extraction. However, in metastatic cancer (stage IV), the knowledge of the current health state of the patient at different times of the disease evolution is crucial for the treatment efficacy^1, 2^. Solid biopsies from metastasis cannot be considered as a monitoring tool as they are highly invasive. Thus, the evaluation of the disease progression is frequently achieved by imaging techniques (MRI, PET and CT scans) and serological tumor markers. In this scenario, the possibility of monitoring the malignancy evolution by simple, low invasive blood extraction (an approach named “liquid biopsy”) with a low-cost device could enable the concept of oncology point of care, where all patients can be adequately screened in a convenient time, at a low cost and without relying on a reference center^3, 4^. Notably, due to the rapid turnover of cancer, tumor-derived markers are continuously released into circulation^5^. These biomaterials include cell-free nucleic acids, proteins, vesicles as well as circulating tumour cells (CTCs) that underwent epithelial to mesenchymal transition, and whose distribution and concentration are correlated with prognosis and metastasis^5^. The analysis of tumour material obtained by liquid biopsies requires highly sensitive assays, which are only emerging within the past few years^3, 4, 6–8^, with CTCs and circulating-tumor nucleic acids as the most investigated targets^9^. Currently, most of liquid biopsy approaches for CTCs are based on the use of the epithelial cell adhesion molecule (EpCAM) antibodies to capture the CTCs and their subsequent characterization with immunostaining or retro-transcription polymerase chain reaction^10–13^. Some methods employ the intrinsic differences in shape, size and/or density of the tumor cells as compared with the normal circulating cells to collect and later recount CTCs by using filters^14–16^, micro-obstacles and gravity^17^, activated surfaces^18–20^ or particles^15, 20–22^. Several CTC detection systems are commercially available and most of them rely on immunomagnetic separation strategies. Notably, CellSearch™ (Veridex)^13^, approved for clinical use in breast, colorectal, and prostate cancer patients, enriches the content of CTCs by using a magnetic ferrofluid containing antibodies against epithelial cell adhesion molecule (EpCAM). CTCs are then separated and treated by common immunohistochemistry methods for the expression of intracellular structural proteins found in epithelial cells such as of cytokeratin- (CK-) 8, 18, and 19. Similarly, in AdnaTest (Qiagen)^23^ the fraction of EpCAM-expressing cells is enriched and isolated using antibody-coated magnetic beads and subsequently identified by real time-PCR. On the other hand, Quadrupole Magnetic Cell Sorter (QMS, Ikotech)^24^ exploits the movement of cells labeled with magnetic nanoparticles through specific antibodies in a magnetic field. Cells can be then collected into low, medium, or high mobility fractions^25^. Other methods employ negative enrichment approaches by depletion of most leukocytes and erythrocytes of the biosample. For example, RossetteSep (StemCell Technologies)^26^ uses a pool of antibodies targeting hematopoietic cells which crosslinks unwanted cells in human whole blood to multiple red blood cells (RBCs), forming immunorosettes^27^. Alternatively, in the EPISPOT (epitelial immunospot) technology^28^ the samples are first enriched in CTCs by using a magnetic CD45 depletion and characterized by positive selection of markers such as by CXCR4, PSA or MUC1.

Microfluidics is a toolbox comprising methods for precise manipulation of fluids at small length scales (μm to mm). Certain properties of microfluidic technologies, such as a rapid sample processing and the precise control of fluids in an assay, have made them attractive candidates to replace traditional experimental approaches in medical and biology research^29^. Microfluidics demonstrates a completely new perspective and an excellent practical way to manipulate cells^30–32^. Microchip devices can be easily prepared using standard microfabrication tools, which lowers cost and simplifies commercialization. Further, microfluidic platforms can be easily integrated with optical techniques^33^ including elastic^34^ and inelastic^35, 36^ light-scattering light-blocking^30^ or fluorescence^29^ methods. This technology allows designing portable and simple devices, operational in ambulatory settings or beside the patient without the need of large equipment and/or specific specialist staff.

Herein, we designed and demonstrated a portable 3D-flow focusing microfluidic device, where all optics are integrated into the chip, for the fluorescence quantification of CTCs in real samples. Notably, our device incorporates two independent interrogation regions for signal correlation analyses which greatly increase the overall detection reliability as compared to traditional single-positive event approaches (e.g., flow-cytometry measurements). This is of outmost importance in applications such as CTCs detection where a very limited number of positive events are expected to be identified in the sample. Although the current body of work^37–39^ provides a glimpse of the outstanding potential of implementing key concepts of hydrodynamic focusing, optical fiber usage, multiple interrogation regions etc. into a device for real life applications, none of these studies neither successfully incorporated these features into an all-integrated chip nor demonstrated its application beyond mere fluorescent beads. In this study, the epithelial cell adhesion molecule, EpCAM^40^, and the receptor tyrosine-protein kinase, HER2^41^, were selected as cell membrane targets to recognise tumor cells of HER2 positive breast cancer^42–44^. Breast cancer malignancy is linked with up to the 30% of the total breast cancers. Further, its correct diagnosis is essential^41^ as different biological features of the disease require specific and differentiated treatments based on monoclonal antibodies or tyrosine kinase inhibitors (TKI) targeting HER2 receptor or its signal transduction pathway (trastuzumab, pertuzumab, TDM1 or Lapatinib)^45–48^. The efficiency of the optofluidic platform was demonstrated on cell lines as well as for a variety of healthy donors and metastatic-breast cancer patients. The results were compared to those of the state-of-the-art diagnostic tools and flow-cytometry.

## Results and Discussion

### Optofluidic chip

**Figure 1:**
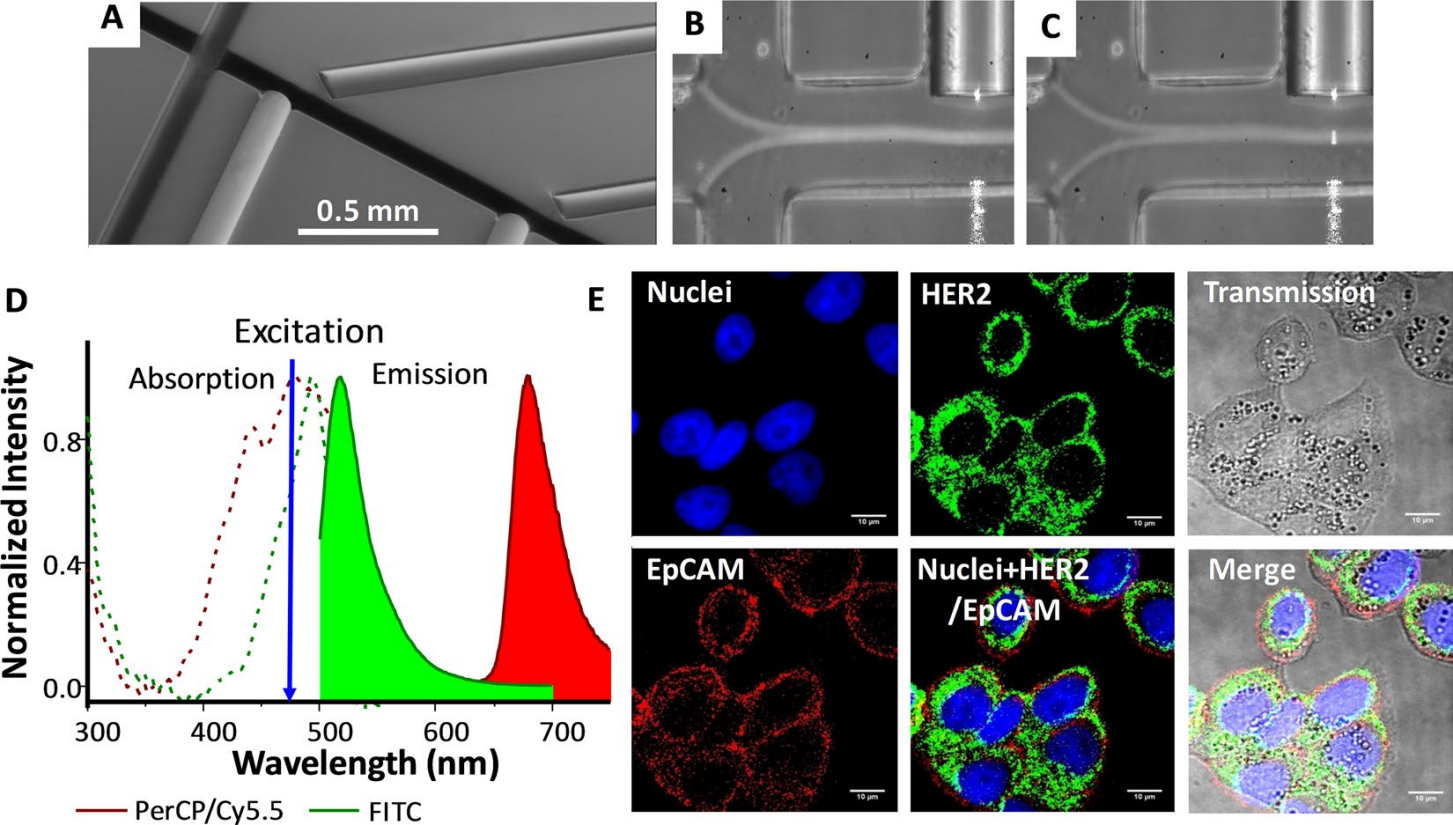
Optofluidic Chip. (**A**) SEM image of the optofluidic chip. (**B** and **C**) Confinement of the sample at the central region of the channel. A blue light path (**C**) is visible only when a cell intersects the pumping beam. Absorption and emission properties of the dyes used to label the antibodies and their performance on the cell membrane. (**D**) Absorption and emission profiles of anti-HER2-FITC (green) and anti-EpCAM-PerCP/Cy5.5 (red) antibodies. (**E**) Laser scanning confocal microscopy images of AU-565 cells stained with anti-HER2-FITC conjugated (green) and anti-EpCAM-PerCP/Cy5.5 conjugated (red) antibodies. Nuclei were stained with DAPI (blue). Scale bar 10 μm.

The microfluidic chip (Fig. 1A and Supplementary Figures S1 and S2) comprises a 3D flow focusing channel for single cell alignment and several integrated optical fibers for excitation and collection. The sample is injected in one of the channels while, simultaneously, a sheath flow is pumped through adjacent channels. The sheath and sample flows do not mix but confine the sample to the central region of the channel (Fig. 1B) due to the low time scales and magnitude of the diffusion rate involved in a typical experiment. Two interrogation zones, placed with a separation of 1 mm, are implemented in the chip. Each of them comprises (i) a pumping fiber (473 nm), which is oriented perpendicularly to the microfluidic channel and focused on the constrained sample, and (ii) a fluorescence collecting fiber (to capture either red or green fluorescence emission). The collecting fibers are oriented at a 45° angle with respect to the excitation source to avoid collecting the light from the pumping source. Single mode fibers are selected for the 473 nm excitation to yield an interrogation volume of the order of the size of the cells (Fig. 1C). Fluorescence emission collection is accomplished by means of two 62.5 μm core high numerical aperture (NA = 0.275) multimode optical fibers, both for the red and the green fluorescence. Such a big core and NA allows obtaining an uninterrupted fluorescence signal from the cells as they flowed through the length of the pumping volume. Within this configuration the system records spectra with acquisition times of 10 μs. It is worth noting that more excitation and detection channels can be easily added along the full length of the microfluidic device. A workflow describing the fabrication methodology of the microfluidic chip is reported in the Supplementary Figure S3.

### Fluorescence signal recording and peak pairing

**Figure 2:**
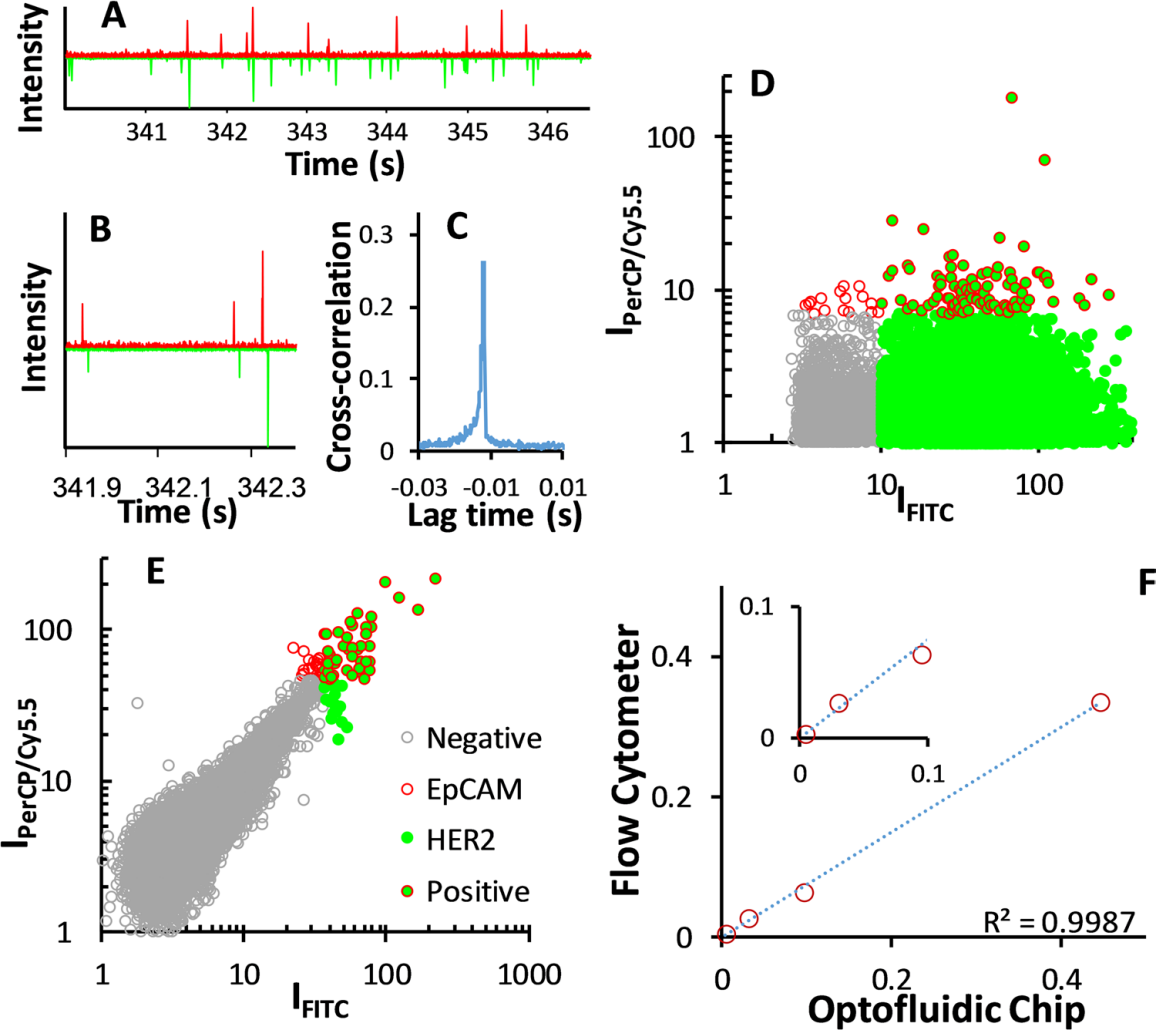
Signal acquisition in the optofluidic chip. (**A**) Short record of the fluorescence signals obtained for the 1:10 sample. (**B**) Cross-correlogram of the green and red fluorescence signals. (**C**) Delay (Δt = −0.0119 s) between the read and the green signals. Results obtained with the (**D**) optofluidic chip and (**E**) flow cytometer for a sample containing 1 AU-565 (HER2+/EpCAM+) per 1000 RAMOS cells (HER2−/EpCAM−). (**F**) Comparison between the optofluidic chip and the flow cytometry cell ratios of 1:1, 1:10, 1:100 and 1:1000 Au-565:RAMOS.

To test the performance of the device we selected two cell lines: (i) AU-565, HER2 positive/EpCAM positive, and (ii) RAMOS, HER2 negative/EpCAM negative. Accordingly, to identify the positive cells we employed anti-HER2 and anti-EpCAM antibodies, labeled with fluorescein isothiocyanate (FITC) and peridinin-chlorophyllprotein cyanin-5.5 (PerCP/Cy5.5), respectively. These fluorescent reporters were chosen based on their absorption properties in the blue spectral range (Fig. 1D), which fit perfectly with the optical fibers excitation wavelength (473 nm), and their well-separated emission bands in the visible range (green, 532 nm, for FICT, and red, 676 nm, for PerCP/Cy5.5). Confocal laser scanning microscopy (Fig. 1E) confirms the efficient staining of AU-565 cells, where HER2-FITC is presented in green and EpCAM-PerCP/Cy5.5 in red. The capability of the optofluidic chip to detect fluorescence-stained cells, even at low concentration, was initially tested on samples consisting of AU-565 (+) mixed with RAMOS (−) cells at different ratios (1:1, 1:10, 1:100 and 1:1000 positive:negative). For comparison, standard flow cytometry was carried out in parallel on the same samples. Figure 2A shows a short record of the fluorescence signals obtained for a sample with 1:10 positive:negative cell ratio. Red signals correspond to the epithelial receptors (EpCAM) while green is assigned to the HER2. The figure displays the normalised intensity of both fluorescent signals. It is worth noting that green emission is larger than the red one due to the higher quantum yield of FICT as compared with PerCP/Cy5.5. Notably, Fig. 2A shows three types of positive events: (i) single green, (ii) single red and (iii) dual green + red. Due to the lack of correlation between apparently random events, the effective recognition of AU-565 cells should be associated only with signal redundancy (i.e., simultaneous green and red positive events, Fig. 2B) as both interrogation zones are exact copies. From the experimental point of view, the two positive fluorescent events can be associated with the same cell by monitoring the time shift between the red and green channels via correlation measurements (Fig. 2C). Specifically, upon acquisition of a positive green signal, the closest red peaks are selected and their elapsed time compared. If the temporal distance between both peaks fits with the adequate time shift corresponding to the travel time of the flowing cells between the two detection areas, the two fluorescent events are considered correlated. Then, results for a given sample can be represented as a function of the respective green and red intensities. Figure 2D and E show the results obtained by measuring a sample of 1:1000 positive:negative cell ratio with the optofluidic chip and a flow cytometer, respectively. Flow cytometry reveals a counting of 43 AU-565 cells (expressing EpCAM and HER2 receptors) and 21163 total cells, evidencing a ratio of 2.04:1000 (minor deviations from the theoretical values can be ascribed to experimental errors). On the other hand, when detecting with the optofluidic chip, 54 malignant and 22765 normal cells were identified. This corresponds to a 2.37:1000 ratio which is in good agreement with the results observed for the standard cytometry. It is worth noting that the optofluidic device produces a considerable larger number of green events than flow cytometry since, under 473 nm pumping, the unspecific cell auto-fluorescence, which mainly takes place in the green part of the spectrum, overlaps with the fluorescence emission of the FITC labelled cells. Similar correspondence was observed, as well, for the rest of measured samples (Fig. 2F and Supplementary Figure S4), validating the feasibility of the method for further applications in real samples.

### Patients and Real samples

**Figure 3:**
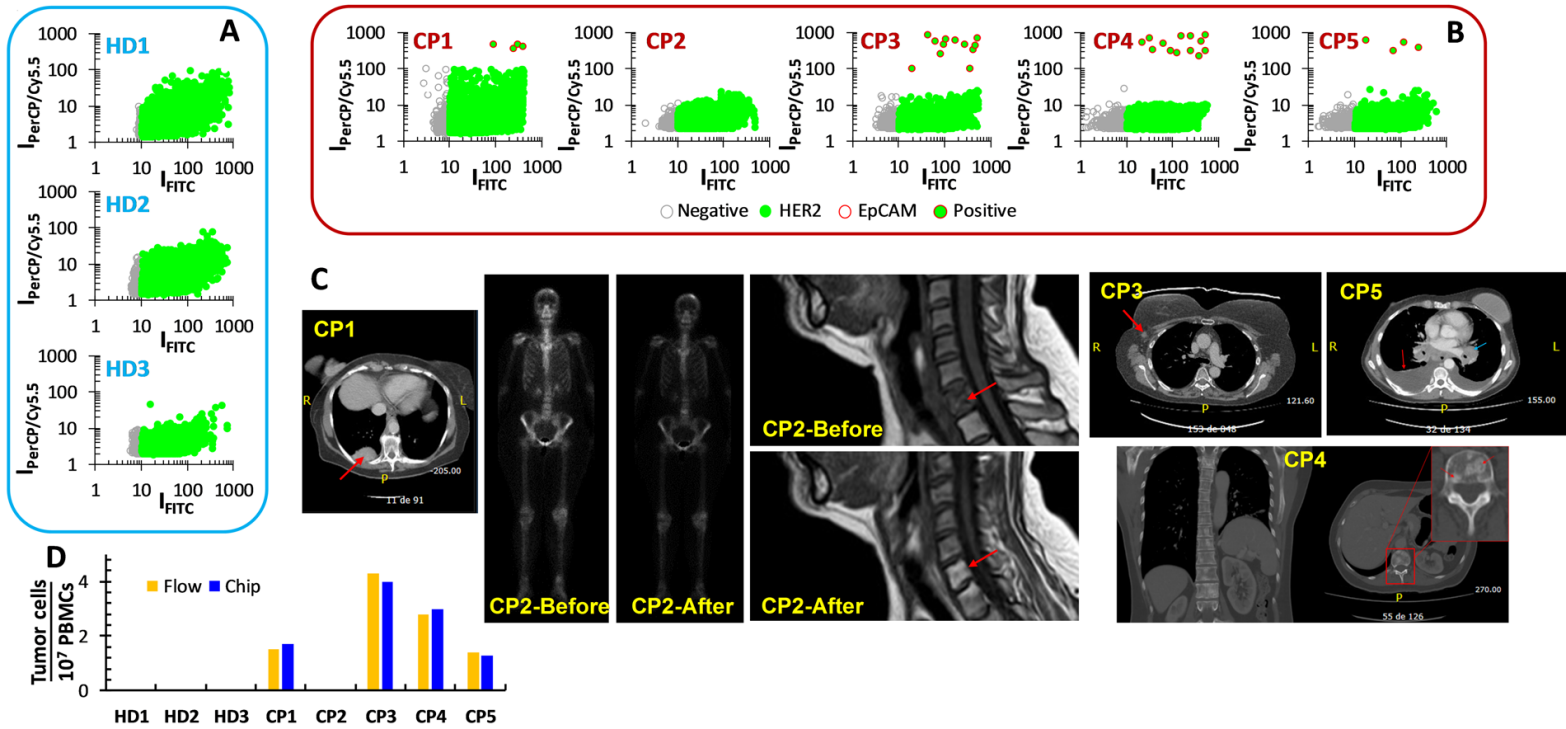
Optofluidic results for blood samples, and computerised tomography of cancer patients at the time of blood extraction. (**A**) 3 healthy donors and (**B**) 5 cancer patients. (**C**) Computerised tomography scans of the cancer patients at the time of blood extraction. CP1, lung metastasis (red arrow). CP2, bone scintigraphy and MRI before (showing a lytic metastasis in C6) and at the time of blood extraction (complete remission). CP3, recently diagnosed and untreated yet metastatic adenopathy (red arrow). CP4, vertebral osteoblastic metastasis showing the current bone progression of the disease (red arrow). CP5, malignant pleural effusion (red arrow) and mediastinal mass (blue arrow). At the time of the study, CP5 had received four cycles of specific treatment. (**D**) Comparison of the results for the blood samples between the flow-cytometry and the optofluidic device.

The presence of CTCs in real blood samples was evaluated by processing and analysing blood from the different healthy donors and the breast cancer patients. Between January and September of 2016, 3 healthy donors and 5 patients were enrolled. All patients were diagnosed with metastatic breast cancer but with secondary tumors in different locations. Their clinical characteristics and cancer evolution at the time of blood extraction are summarised in Table 1. Figure 3 includes the computerised tomography scans of the patients at the time of blood extraction. Previous data had been also added to patient 2 as she is currently in complete remission. Personal and clinical data were recorded according to standard clinical procedures. All patients provided written informed consent before study enrollment. The study was approved by the local Ethics and Clinical Research Committee.

**Table 1:** Clinical characteristics and the state of cancer progression at the time of blood extraction.

Patient	Age (yr.)/Sex	ER/PR	Metastasis	Disease Status
CP1	68/F	Positive	Visceral	Stable
CP2	41/F	Negative	Bone	Complete remission
CP3	59/F	Positive	Lymph node	Progression
CP4	44/F	Negative	Visceral	Progression
CP5	49/F	Positive	Bone	Progression

F, Female; ER, estrogen receptor; PR, progesterone receptor.

To reduce the initial volume of the real sample (8 mL), only the peripheral blood mononuclear cells (PBMCs) were collected, stained, and run into flow cytometry and the optofluidic chip. Blood from healthy donors, with no CTCs, was first run to establish a threshold which identifies the intrinsic level of positive events ascribed to normal PBMCs (Fig. 3A and Supplementary Figure S5). Contrary to what observed for cellular lines, no spurious EpCAM positive events were observed by both flow cytometry and optofluidics. On the other hand, the number of HER2 positive events was notably larger. In the case of the flow-cytometer, such increase in the number of HER2 positives can be ascribed to expression, although low, of HER2 receptors in the NK/granulocyte population^49^. For the optofluidic chip, this biological factor is further combined with the additional background cell auto-fluorescence which is collected at the green channel. Once defined the thresholds associated with positive events for both green (antiHER2) and red (antiEpCAM) labels, flow cytometry and optofluidic measurements were performed on blood samples from cancer patients (Fig. 3B and Supplementary Figure S5). CTCs were quantified in similar amounts, with both methods (Fig. 3D), for four of the five patients but with one presenting none (CP2). These data correlate well with those obtained with conventional diagnosis at the sample extraction (Table 1 and Fig. 3C). First, samples which show low CTC counts (CP1 and CP5) present a stabilised lung metastasis (CP1) and a malignant pleural effusion and mediastinal mass (CP5), which is still in progression but after four cycles of specific treatment. In contrast, CP4 presents an osteoblastic metastasis in progression while the patient with a larger number of CTCs (CP3) was recently diagnosed with progression and is already initiating treatment. Notably, we had no evidence of CTCs in the blood of CP2, who is currently in a complete remission of the previously diagnosed C6 and other bone lytic and visceral metastasis. By considering the epithelial-mesenchymal transition in cancer metastasis^50^, these results suggest that CTCs in a metastatic cancer patient cannot be restricted to those with epithelial lineage. In fact, by analysing the HER2 positive signals from the healthy donors and the cancer patients, it stands out that patients in progression (CP3, CP4 and CP5) have similar HER2 patterns similar which differs from those of healthy donors. Conversely, the samples of the patient in complete remission (CP4) reveal a pattern similar to those of the healthy patients, while CP1 (stable status) displays a pattern which lies in between those of healthy and CP3-5 patients. Although this observation is preliminary and requires a larger number of patients and healthy donors for confirmation, it points toward the use of another marker to characterise the disease and, probably, in its earlier stages.

## Conclusions

In summary, we designed and fabricated a low-cost hydrodynamic optofluidic chip with all integrated optics. This chip can confine the sample fluid to screen single cells, therefore providing an efficient tool to quantify the number of CTCs in a given liquid sample. To evaluate the system, we selected the HER2 positive breast cancer, a widely spread and aggressive tumor which requires of special surveillance. The system was engineered with two detectors to redundantly identify positive CTCs by the simultaneous identification of signal belonging to two membrane receptors, HER2 and EpCAM. Results acquired in real blood samples from metastatic HER2 breast cancer, at different metastatic degree and progression, show that the optofluidic device can successfully define the health condition of the patient. The results well correlate with those from flow-cytometry as well as with the conventional imaging and serological data obtained from the patient before blood extraction. Joining a list of next-generation diagnostics (MALDI-TOF, lateral-flow, PCR), our proof-of-concept study aims to build a platform for the early identification and monitoring of tumors that may result in improved patient outcomes and decreased treatment toxicities. This sensing platform can be easily extended to the investigation of other tumors by employing different selective antibodies. Finally, with a growing population of patients at risk of developing cancer, a highly sensitive, nonsurgical, nonradioactive method for repeated monitoring will be clinically useful.

## Methods

### Fabrication of PDMS microfluidic chips

3” Si wafers were used as a substrate to produce the SU8 (SU8-2150, MicroChem Corp) structures. Prior to SU8 spin coating, the Si wafer is thoroughly dehydrated on a hot plate at 200 °C for two hours. An adhesion promoter (OmniCoat, MicroChem Corp.) was then applied over the bare Si surface and then baked on a hotplate at 200 °C for one minute. The wafer was kept at 100 °C on a hot plate to avoid any hydration of the substrate. SU8 masters were fabricated employing a standard technique widely used during master construction for PDMS. Microfluidic chip pattern was engraved using a submicron resolution UV laser lithography DWL 66fs from Heidelberg Instruments on a 4 inches chromium photomasks. For a single chip elaboration two molds were constructed, one containing the structures for the base of the chip, microfluidic channels and grooves for the optical fibers, and another one from which the channels of the cover, devoted to vertical sample flow focusing, were cast. The width of the optical fibers grooves was set during the photomask elaboration at 120 µm (5 µm below the diameter of the stripped optical fiber) to ensure a proper grip between the fiber cladding and the PDMS. All channels were 120 µm in height and had a width of 100 µm. Care was put during the construction of the photomask so that, after the realization of the SU8 master, the channels containing the pumping fibers and the collection fibers would be aligned in such a way that the axis of the fibers would intersect exactly in the middle of the microfluidic channel once they were positioned. For the spin-coating process, 2 mL of SU8 were dispensed over a dehydrated 3 inches Si wafer and then spin-coated at 2800 RPM for 40 seconds with a 500 RPM/s ramp. These parameters allowed obtaining a SU8 thickness of approximately 120–130 µm; the required to prevent fibers from protruding from the PDMS groove. The wafer with the SU8 layer was then soft-baked for 1 h at 100 °C. Once the soft-bake finished and the solvent from the SU8 was completely evaporated, an edge bead removal (EBD) of the SU8 spin coated layer was performed. This process allows the photomask to be in close contact with the baked SU8 exposed surface during the UV exposure, minimizing UV light diffraction effects in the patterned mask contours. The UV exposure of the SU8 was performed with a mercury discharge lamp with mask aligner mounting (MG 1410, Karl Suss). The optimal exposure time was found to be 4.5 seconds for a density power of 124 mW/cm^2^ for a reference wavelength of 405 nm. After the SU8 has been exposed it follows a post exposure bake at 65 °C for 5 min and 100 °C for 10 min. The hot plate is then switched off and allowed to cool down to room temperature. The Si wafer is then developed in PGMEA solvent (AZ® EBR Solvent) multiple times until the SU8 structures are exposed and no uncrosslinked SU8 residue is visible on the Si wafer. An oxygen plasma exposure was applied to the finished master to eliminate the exposed Omnicoat layer and leave a cleaner Si surface. A reactive-ion etching (RIE) (PlasmaPro NGP80, Oxford Instruments) was used for that purpose and the conditions were: power: 100 W, oxygen flow rate: 35 sccm, pressure: 100 mTorr and time: 1 min.

### PDMS bonding and optical fiber positioning

The SU8 masters were placed at the bottom of an aluminum foil container with the face with the SU8 structures pointing upwards and the degassed PDMS (Sylgard 184, Dow Corning) was later poured into the container and left to cure at 80 °C for 2 h. After curing, the PDMS slabs were then thoroughly rubbed with optical cleaning tissues soaked in acetone and rinsed with ethanol. The PDMS surfaces were then irreversibly bonded together exposing the surfaces to a RIE generated oxygen plasma under the following conditions: power: 10 W, oxygen flow rate: 50 sccm, pressure: 80 mTorr and time: 20 s. Figure 1 shows an ESEM image of both interrogation zones with pumping and collecting fibers inserted for illustration purposes. A total of four single-ended FC connectorized optical fiber patch cords were inserted in a single chip. To ensure a proper illumination and a clean diverging Gaussian beam in the interrogation zone, the fibers were cleaved and later observed under the microscope to verify a clean cut of the face of the fiber before inserting them by hand in the dedicated grooves entrances located at both sides of the chip. Likewise, flat and homogeneous fiber cleaves guaranteed an optimal light harvesting for the collection fibers. Each groove end was wetted with a droplet of ethanol which entered inside the groove by capillarity. This reduced the friction and helped during the process of fiber insertion.

### Sample measurement with the optofluidic chip

The optofluidic chip was placed under an inverted microscope (IN480TC-FL, AMScope) to obtain clear images of the focusing of the fluid. Two pressure controllers (OB1-mk3, Elveflow) were employed to pump the fluid inside the chip; one channel for each of the four sacrificial fluids -two for vertical focusing and two more for horizontal focusing- and one channel for the sample flow. The independent treatment of each input flow allowed not only to adjust the area of the cross-section of the centrally focused flow but also the position of the focused flow within the microfluidic channel with respect to the walls of the central microfluidic channel. Typical pressure values for the device were 35–40 mbar for the sheath flows and 10 mbar for the sample flow. That led to flow rates for the sample flow of ca. 20 μl/min. The upper limit for the sample flow rate is set by the maximum pressure deliverable at the dedicated channel by the pressure controller (200 mbar). At this maximum pressure, the resulting flow rate is expected to be ca. 150–200 μl/min. The pressure at the remaining channels (with a higher maximum pressure set point) should be then increased accordingly to maintain the focused sample geometry. At these pressure conditions, the mean velocity in the sample flow channel is estimated to be ca. 0.2 m/s. Importantly, the data acquisition card would not represent a bottleneck while working at full sampling speed (4 × 10^5^ S/s). Under these circumstances, we would record approximately 20 points for each event (a passing cell in the interrogation region) for an interrogation region of approximately 10 μm across.

The pumping light for both independent interrogation zones was generated by a laser diode (473 nm 06-01 series, Cobolt) coupled to a 50:50 1 × 2 optical fiber splitter (FC488-50B-APC-1, Thorlabs). Each splitter end was mated to both single mode input optical fibers of the chip. Typical pumping powers inside the microfluidic channel during the measurement process were of the order of 10 mW. The two-output fluorescence collection multimode patch fibers of the microfluidic chip were directly connected to two photomultiplier tubes (R928, Hamamatsu) enclosed in an aluminum housing (PXT1/M, Thorlabs) using an FC connector. To avoid possible high pumping light intensities from reaching the photomultiplier (originated mainly from scattered light in the cells) two 500 nm cut-off wavelength longpass filters (FELH0500, Thorlabs) were located in the aluminum housing between the optical fiber tip and the photocathode. Additional bandpass filters (86–988 and 33–330 from Edmund Optics) were placed next to each longpass filters to allow the selected transmission of light at each photomultiplier detector. Both photomultiplier tubes were directly connected to an oscilloscope (TDS 1012B from Tektronix) to visualise fluorescence bursts simultaneously while the measuring process was taking place. The internal resistance of the oscilloscope (1 M Ω) was used as the output impedance. A two-channel home-made voltage follower with a saturation voltage of 9 V was then applied to the voltage signal from the oscilloscope and a data acquisition card (NI USB-6212 BNC, National Instruments) was used in order acquire the signal data from both channels. The low impedance in the voltage follower prevented from ghosting to appear in one of the channels, especially at high sampling rates in the DAQ. To minimise any noise from electronic equipment coaxial cables were employed to connect all the electronic components of the setup. Data acquisition from the signal was accomplished by using a Lab-VIEW program that allowed to simultaneously saving the data from the two channels to be later treated using Scilab software for correlation measurements of the two signals.

### Sample measurement with the flow-cytometer

Flow cytometry of the samples was carried out in a NovoCyte Flow Cytometer (from AceaBiosciences), equipped with a 488 nm excitation laser and two different filter, 530/30 nm for FITC fluorescence collection and 675/30 nm for red PerCP/Cy5.5 collection. A negative (non-labelled) sample was first analyzed in the cytometer in order to set a threshold for the intrinsic fluorescence of cells in both colors. Then, all events (cells) showing intensities higher than the established threshold are considered as positive-stained cells. Plotting FITC Vs PerCP/Cy5.5, double stained cells are shifted to high intensities while negative cells are located at lower intensities. Cytometric data were analysed with NovoExpress and FloJo VX software.

### Cell Culture

AU-565 cancer cells derived from mammary breast expressing HER2 and EpCAM receptors, and RAMOS, HER2, EpCAM negative, were obtained from the American Tissue Culture Collection (ATCC, Manassas, VA, USA) and cultured in RPMI media supplemented with 10% fetal bovine serum, at 37 °C in a 5% CO_2_ atmosphere.

### Fluorophores characterization

Absorption and emission profiles for FITC and PerCP/Cy5.5 conjugates were collected with a UV-visible spectrophotometer Evolution 201 (ThermoScientific) and Cary Eclipse Fluorescence Spectrophotometer (Agilent Technologies).

### Cell Labelling for microscopy analysis

AU-565 cells were harvested from culture dishes with trypsin/EDTA 0.25% and seeded in 8-well µ-plate Ibidi at 1 × 10^4^ cells/well, for 24 h at 37 °C in a 5% CO_2_ atmosphere. Following day, cells were washed with PBS and fixed with 4% paraformaldehyde in PBS for 30 min. Then, cells were treated with 1% NP40 in PBS for 15 min at 37 °C. 2.0 µg/mL of mouse anti-human her2 FITC-conjugated antibody (Abcam) and 2.0 µg/mL of mouse anti-human EpCAM PerCP/Cy5.5-conjugated antibody (Abcam) were added to AU-565 cells for 1 h of incubation at 37 °C. Finally, cells were washed three times with PBS and 100 nM DAPI was added for nuclei staining. Cells were analyzed by laser scanning confocal microscopy using 488 nm excitation laser and 540/30 nm and 650LP nm detection filters, to collect fluorescence from FITC and PerCP/Cy5.5, respectively. DAPI fluorescence was collected with epi-fluorescence filters and images were processed with ImageJ software.

### Cell Labelling for microfluidics and flow cytometry

Cells were harvested from culture dishes with EDTA 0.25% PBS dissociation buffer and 1 × 10^6^ cells were placed in suspension in PBS 10% FBS. For each 100 µL of cell suspension, with the appropriate AU-565:RAMOS ratio, 4 µL of mouse anti-EpCAM-PerCP/Cy5.5 and 10 µL of mouse anti-HER2-FITC were added. Then, cells were washed by centrifugation at 170 g for 5 min and suspended in cold-PBS. Samples were measured in the optofluidic device and the flow-cytometer.

### Clinical samples

All experiments were approved by Ethics and Clinical Research Committee of the HM Hospitales Group (Madrid, Spain, www.hmhospitales.com). All experiments were performed in accordance with relevant guidelines and regulations approved by Ethics and Clinical Research Committee of the HM Hospitales Group (Madrid, Spain, www.hmhospitales.com). All personal and clinical data were recorded in accordance with relevant guidelines and regulations approved by Ethics and Clinical Research Committee of the HM Hospitales Group (Madrid, Spain, www.hmhospitales.com). All specimens were obtained with informed consent from all participants. 8 mL of blood was drawn in 10 mL vacutainer tubes containing EDTA and processed in the first 24 h. Results for CTCs were linked to clinical data.

### Preparation of clinical samples

Blood samples from cancer and healthy patients were obtained from the Servicio de Oncología Clínica of the HM Hospital Universitario Torrelodones-Madrid. Peripheral blood mononuclear cells (PBMCs) were isolated from the whole blood using Ficoll-Paque PLUS (purchased from GE Healthcare Life Science). 15 mL of Ficoll solution was added to a 50 mL Leucosep Centrifuge tubes (Greiner Bio One) and blood was disposed as a layer onto Ficoll. Samples were centrifuged at 400 g for 40 min at 18 °C, and the resulting PBMCs layer was separated from the rest of phases. PMBCs were washed twice in 10 mL HBSS by centrifugation at 100 g for 5 min and finally suspended in RPMI-1640 supplemented with 10% FBS, until use. Then, 1 × 10^7^ cells were pelleted and placed in 100 µL PBS 10% FBS and 4 µL of anti-EpCAM-PerCP/Cy5.5 and 10 µL of anti-HER2-FITC were added. After 1 h at RT, cells were washed by centrifugation at 500 g for 5 min and suspended in cold-PBS. Same procedures were following for the measurement of clinical samples in the optofluidic device and the cytometer, as mentioned above.

## Electronic supplementary material

Supplementary Information
