# Supplementary material for: Optofluidic device for the quantification of circulating tumor cells in breast cancer
Source: Sci Rep. 2017 Jun 16;7:3677. doi: 10.1038/s41598-017-04033-9 (PMC5473916; doi:10.1038/s41598-017-04033-9)
Supplement: Supplementary file 1 — Supplementary Information [file 41598_2017_4033_MOESM1_ESM.pdf]

SUPPLEMENTARY INFORMATION

Optofluidic device for the quantification of circulating tumor cells in breast cancer

Eric Pedrol[1], *Manuel Garcia-Algar*[2], *Jaume Massons*[1], *Moritz Nazarenus*[2], *Luca Guerrini*[2], *Javier Martínez*[1], *Airan Rodenas*[1], *Ana Fernandez*[2], *Magdalena Aguiló*[1], *Laura G. Estevez*[3,4], *Isabel Calvo*[3,4], *Ana Olano*[5], *Eduardo Garcia-Rico* [3,4,5,]*, *Francesc Díaz* [1,]* & *Ramon A. Alvarez-Puebla.*[2,6,]*

[1]Física i Cristal•lografia de Materials i Nanomaterials and EmaS. Universitat Rovira i Virgili, Carrer Marcel•lí Domingo 1, 43007 Tarragona (Spain). [2]Departamento de Química Física e Inorgánica, Universitat Rovira i Virgili, Carrer Marcel•lí Domingo 1, 43007 Tarragona (Spain). [3]Fundacion de Investigacion HM Hospitales, San Bernardo 101, 28015 Madrid (Spain). [4]Centro Integral Oncologico Clara Campal (CIOCC), Oña 10, 28050 Madrid (Spain). [5]Department of Medical Oncology, Hospital Universitario HM Torrelodones, Castillo de Olivares s/n, 28250 Torrelodones (Spain). [6]School of Medicine, San Pablo CEU, Calle Julián Romea, 18, 28003 Madrid (Spain). [7]ICREA, Passeig Lluís Companys 23, 08010 Barcelona (Spain).

Correspondence and request for materials should be addressed to F.D. (email: f.diaz@urv.cat), E.G.R. (email: egarcia@hmhospitales.com), or R.A.P. (email: ramon.alvarez@urv.cat).

E.P. and M.G.A. equally contributed to this work.

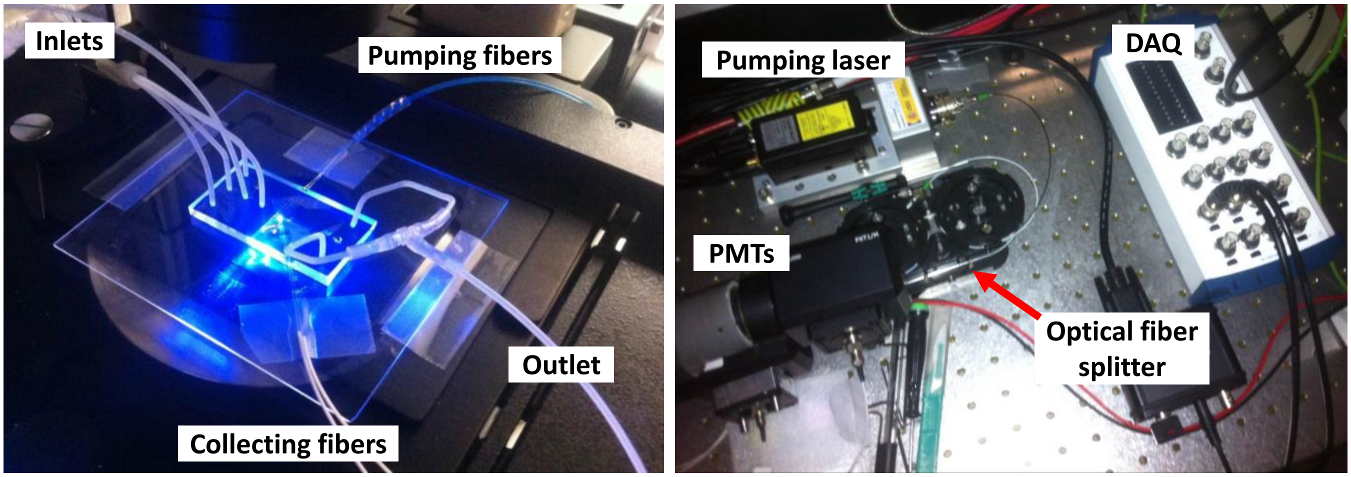

Figure S1. Optical images of the optofluidic device setup and its components.

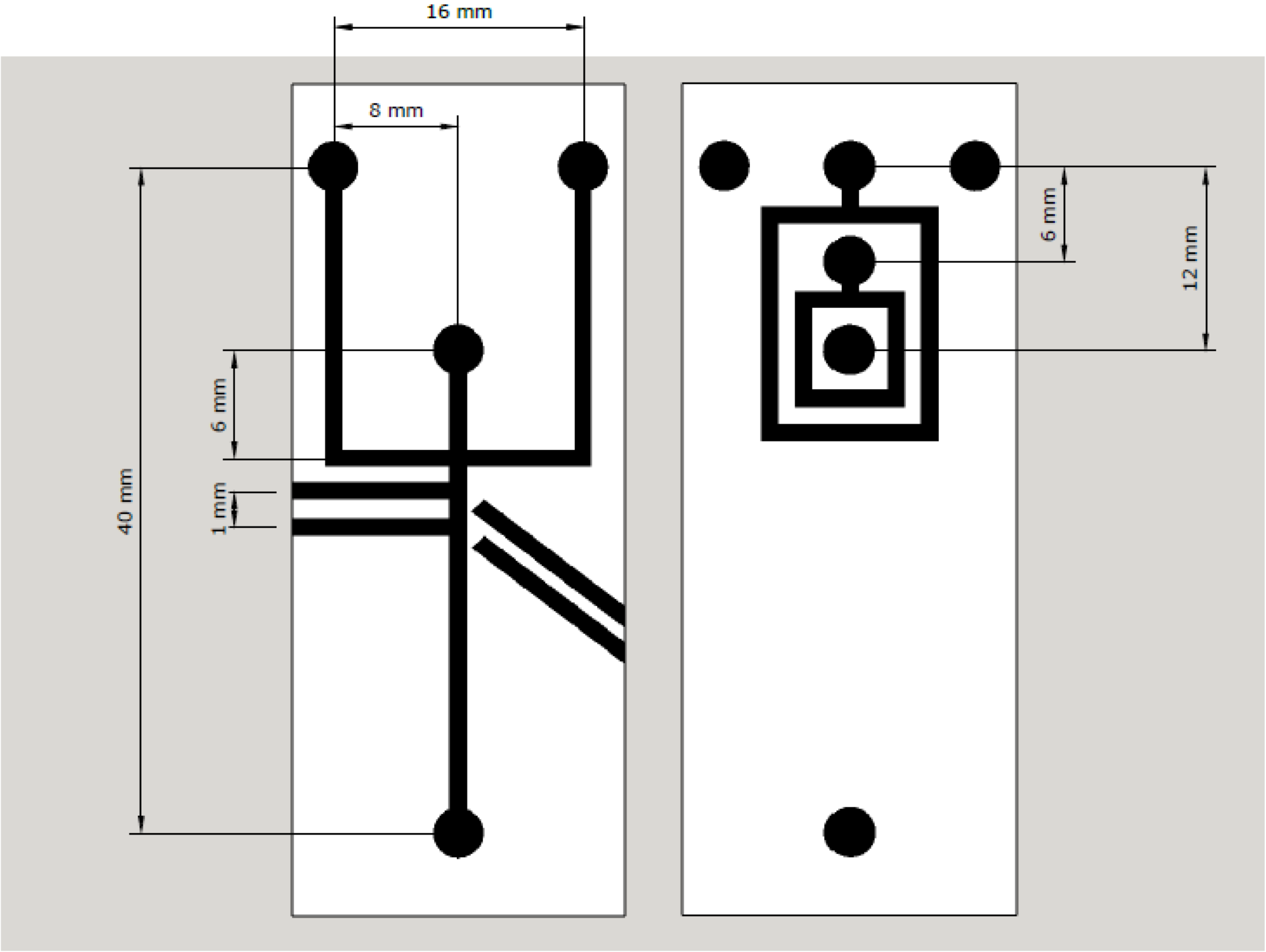

Figure S2. Schematic design of the microfluidic chip.

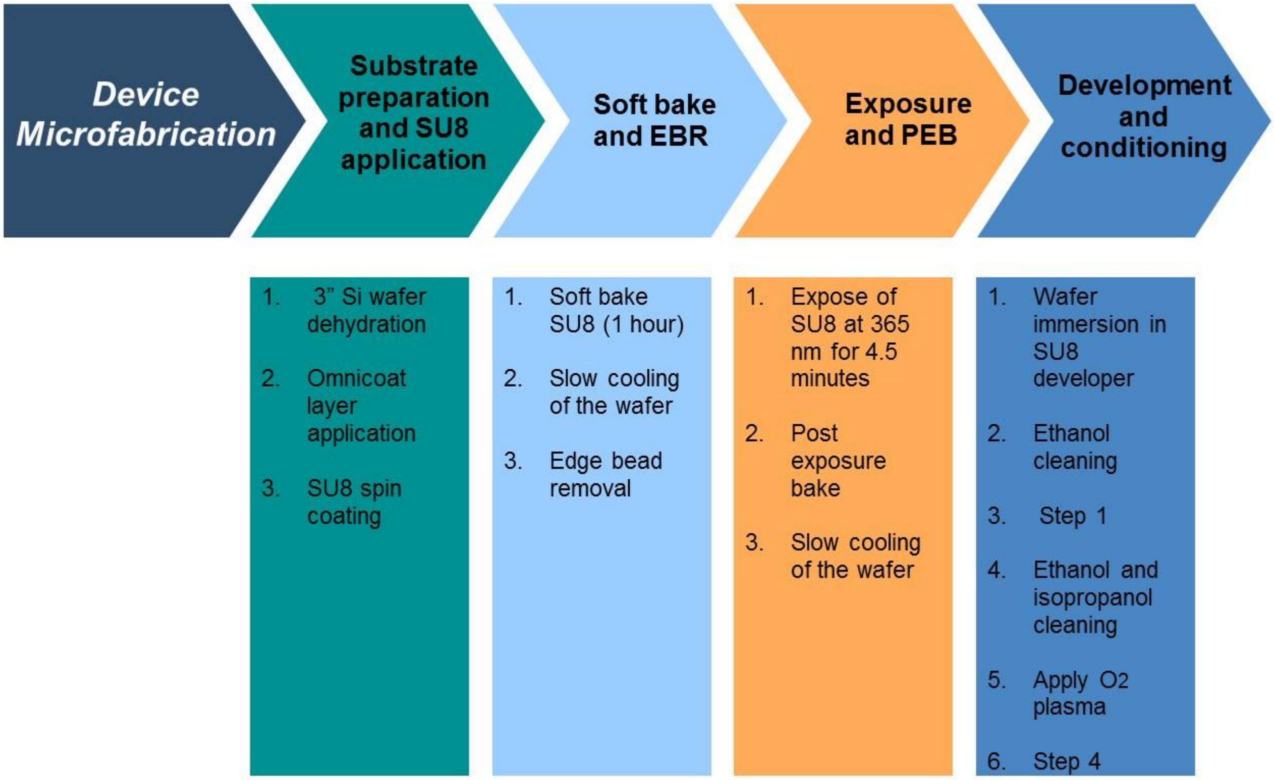

Figure S3. Schematic workflow of the microfluidic chip fabrication.

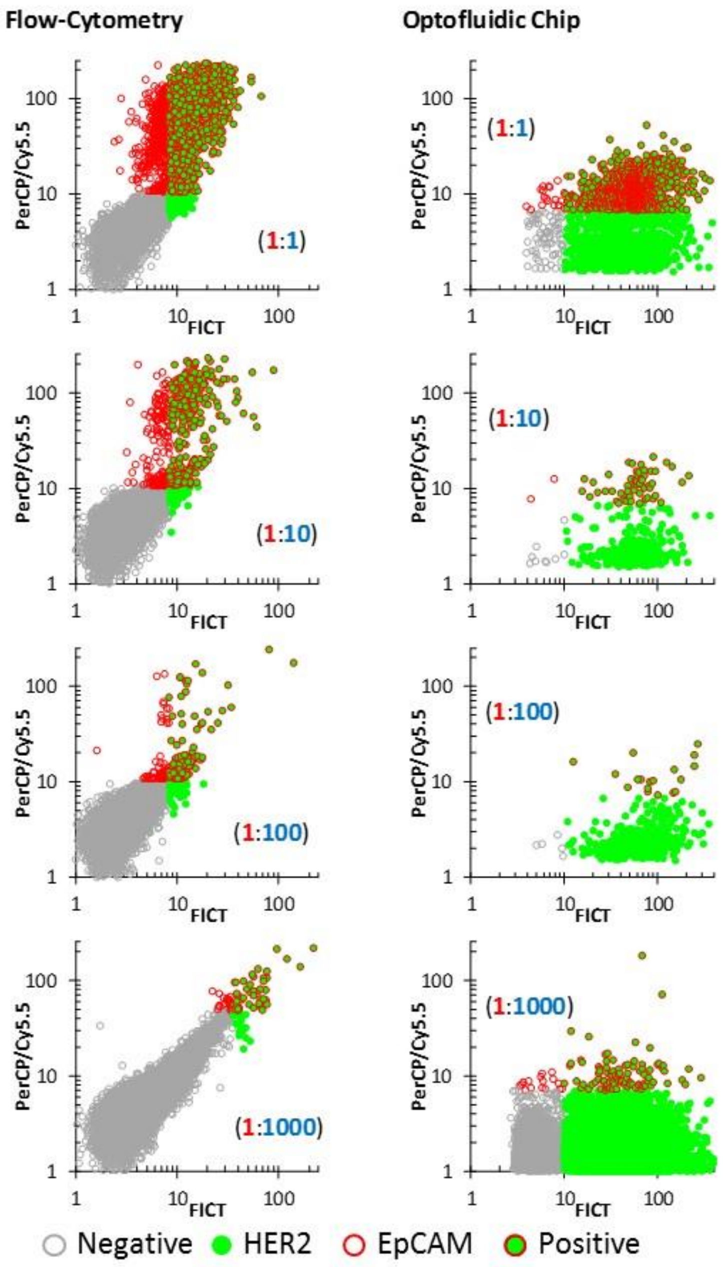

Figure S4. Results obtained with the flow cytometer and the optofluidic chip for cell ratios of 1:1, 1:10, 1:100 and 1:1000 Au-565:RAMOS. Substantial differences between the green and red signals from the flow-cytometry and our optofluidic device are mainly due to the different excitation wavelengths (488 nm in the flow cytometer, and 473 nm in the optofluidic device). In fact, sample cells present more autofluorescence at 473 nm than 488 nm which overlaps with the green fluorescence. In the case of the red signal (EpCAM), ratios are similar; however, the number of cells monitored in each experiment was different.

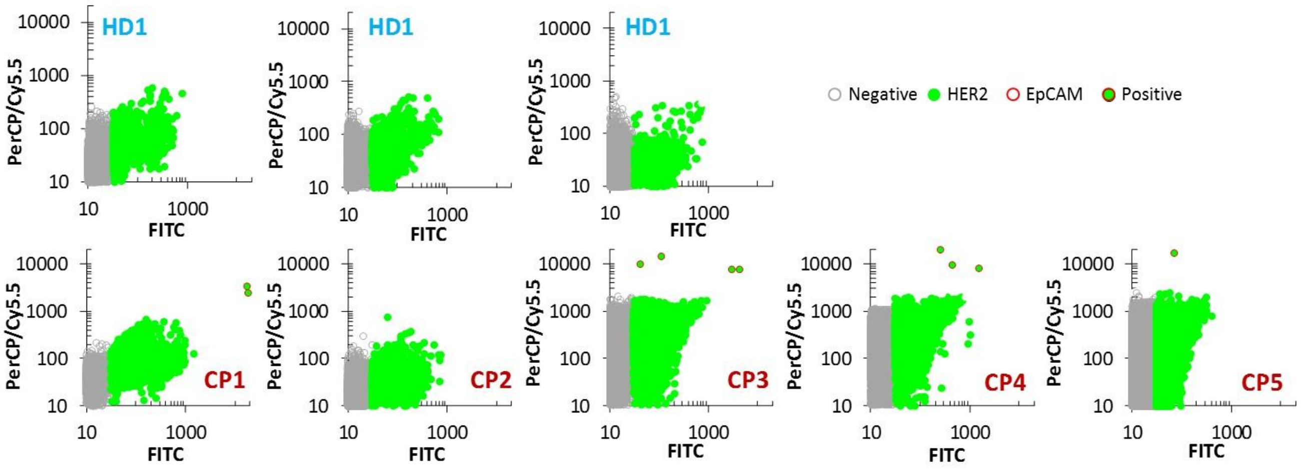

Figure S5. Results obtained with the flow cytometer for the blood samples from healthy donors and cancer patients.
